# Adaptive Linear Quadratic Attitude Tracking Control of a Quadrotor UAV Based on IMU Sensor Data Fusion

**DOI:** 10.3390/s19010046

**Published:** 2018-12-22

**Authors:** N. Koksal, M. Jalalmaab, B. Fidan

**Affiliations:** Department of Mechanical and Mechatronics Engineering, University of Waterloo, 200 University Avenue West, Waterloo, ON N2L 3G1, Canada; nkoksal@uwaterloo.ca (N.K.); mjalalma@uwaterloo.ca (M.J.)

**Keywords:** quadrotor UAV, adaptive linear quadratic tracking, indirect estimation, least squares estimation, sensor fusion, Kalman filter

## Abstract

In this paper, an infinite-horizon adaptive linear quadratic tracking (ALQT) control scheme is designed for optimal attitude tracking of a quadrotor unmanned aerial vehicle (UAV). The proposed control scheme is experimentally validated in the presence of real-world uncertainties in quadrotor system parameters and sensor measurement. The designed control scheme guarantees asymptotic stability of the close-loop system with the help of complete controllability of the attitude dynamics in applying optimal control signals. To achieve robustness against parametric uncertainties, the optimal tracking solution is combined with an online least squares based parameter identification scheme to estimate the instantaneous inertia of the quadrotor. Sensor measurement noises are also taken into account for the on-board Inertia Measurement Unit (IMU) sensors. To improve controller performance in the presence of sensor measurement noises, two sensor fusion techniques are employed, one based on Kalman filtering and the other based on complementary filtering. The ALQT controller performance is compared for the use of these two sensor fusion techniques, and it is concluded that the Kalman filter based approach provides less mean-square estimation error, better attitude estimation, and better attitude control performance.

## 1. Introduction

Unmanned aerial vehicle (UAV) systems, particularly quadrotor UAV systems, have been popular in various autonomous surveillance and transportation applications in recent years. Robotics and control researchers have been interested in improving quadrotor UAV systems with regard to path planning, tracking, stability and autonomous motion capability in simultaneous localization and mapping (SLAM) tasks for difficult missions such as defense patrol duties, agricultural activities, surveillance, and rescue [1,2,3,4,5,6].

In the literature, various control approaches have been proposed for quadrotor UAV systems. For attitude tracking control and stabilization, researchers have developed solutions such as quaternion-based feedback control for exponential attitude stabilization [5], robust adaptive attitude tracking control [7], robust attitude control for uncertain quadrotors with proportional-derivative (PD) controller combined with a robust compensator [8], robust nonlinear design under uncertainties and delays [9], and fractional sliding modes based attitude control [10].

One of the main control interests for quadrotors UAV is optimization of time and energy (battery) consumption by designing optimal path planning and optimal tracking control. For such optimal attitude tracking, Ref. [1] has designed a linear quadratic regulation (LQR) based attitude stabilization. For solving a more general form of the same problem under wind gust disturbances, a switching model predictive attitude controller is developed in [11]. Ref. [12] presents $L_{1}$ optimal robust tracking control to compensate persistent disturbances in translational and rotational (attitude) dynamics.

Linear-quadratic (LQ) based optimal control frameworks constitute a systematic toolset for calculating ideal control gains with guaranteed system stability under LQ design conditions. LQ-based control schemes provide robust and precise steady-state tracking while the performance index (quadratic cost function) adjusts optimality trade-off between tracking/regulator performance and battery consumption. A particular the LQ-based control approach is infinite-horizon optimal regulation based on linear time-invariant (LTI) models. This approach is widely used in real-time applications since its solution does not have computational complexities for obtaining constant state-feedback control (Kalman) gains by solving the algebraic Riccati Equation (ARE). The infinite-horizon LQR has been mostly used in many earlier works as studied for the quadrotor UAV in [1] for attitude state regulation and stability.

On the other hand, linear-quadratic tracking (LQT) problems have gained less attention compared to LQR problems, since time-varying reference trajectories lead to further analysis and computational complexities. LQT control schemes typically consist of two state-feedback and feed-forward terms. The state-feedback terms guarantee system stability by state-feedback (Kalman) gains which are calculated offline solving differential Riccati equation (DRE). The feed-forward terms provide optimal tracking of time-varying bounded reference trajectories utilizing the differential auxiliary vector signal equation. In practice, computational complexities arise because of the time variations in the feed-forward terms. Accordingly, the literature on LQT control design and applications on real-time systems is limited. Ref. [13] presents an offline solution to the infinite-horizon LQT problem by solving the feed-forward term based on calculating the initial condition of the auxiliary vector signal. The authors present a real-time implementation of this solution on flexible beams system in [14]. Other than the classical solution, Ref. [15] presents an online reinforcement learning algorithm to solve LQT problem without requiring the knowledge of the system drift dynamics or the command generator dynamics.

Regarding LQT of quadrotor UAV systems, Ref. [16] presents a finite-horizon LQT control design with time-varying control gains which are calculated solving offline discrete time matrix Riccati equations for the linearized full dynamics of the quadrotor UAV. Consideration of finite-horizon LQT with known boundary conditions at the initial and final time instants prevents the computational complexity issues with implementation of this design. However, in many practical cases, including the cases considered in this paper, since the boundary conditions are unknown, infinite-horizon LQT needs to be considered for designing an alternative optimal linear tracking controller.

In this paper, by the motivations of LQ-based optimal control advantages as stated above and lack of infinite-horizon LQT control schemes with their applications on real-time systems in literature, we present an infinite-horizon LQT control design, its practical solution and its experimental validation on the real-time quadrotor UAV with inertial parametric uncertainties and Inertial Measurement Unit (IMU) sensor noises. Furthermore, to improve robustness against parametric uncertainties, the presented LQT control design is combined with an adaptive parameter identification (PI) scheme based on least-squares (LS) estimation. Combining the LQT control design and the PI scheme, an adaptive LQT (ALQT) control scheme is developed for optimal attitude tracking of quadrotor UAVs, with reduced tracking error and battery consumption.

Reliable attitude estimation is one of the main challenges for quadrotor UAV tracking control. Euler angles (ϑ, ϕ, and ψ) and Quaternions are two common types of attitude representation for UAV systems. IMUs, formed by 3-axis inertial sensors of gyroscopes, accelerometers and magnetometers, measures angular velocities, linear accelerations and the Earth’s magnetic field. Ideally, accelerometer measurements or numerical integration of angular velocities of gyroscopes should be enough for ideal sensors to determine attitude angles. However, in real-world conditions, individual usage of these sensors is not sufficient to determine attitude angles due to large amounts of system noise, drift errors and vibrations.

To obtain fast and accurate attitude states, sensor fusion techniques have been applied to IMU measurements, including wide ranges of complementary filters [5,17,18,19,20,21,22,23,24] and Kalman filters [23,24,25,26,27,28,29,30,31,32,33,34,35]. A complementary filter typically combines accelerometer output for low-frequency attitude estimation with integrated gyroscope output for high-frequency estimation. Complementary filters are computationally less demanding, and, due to their simplicity and efficiency, these filters are still used for attitude estimation. A variety of complementary filters has been used to estimate attitude quaternions [19,20,21] or Euler angles for relatively small roll and pitch aerial vehicle angles [5,22,23,24]. Complex rotations of simultaneous roll, pitch and yaw angles require nonlinear complementary filter fusion techniques [36].

Kalman filter is an optimal recursive estimation scheme that uses a system’s dynamic model, known control inputs, and multiple sequential measurements from sensors to form an estimate of the system states fusing prediction and measurement online [25,26,27,28]. The extended Kalman filter (EKF) is developed for nonlinear system state estimation and has been widely used for real-time UAV systems for Euler angle based attitude estimation [23,24,29,30] as well as quaternion based attitude estimation [31,32,33,34,35]. Unscented Kalman filter (UKF) [37,38,39] and adaptive Kalman filter (AKF) [40] are other widely used sensor fusion algorithms. In [30], a novel Kalman filter algorithm is proposed, which consists of an EKF and an inverse Φ-algorithm in a master–slave configuration to estimate reliable angular acceleration signals by fusing IMU sensor data. In [35], it is shown that, even for applications with strong real-time constraints, EKF can properly estimate the UAV attitudes, even in the presence of data loss.

**Figure 1:**
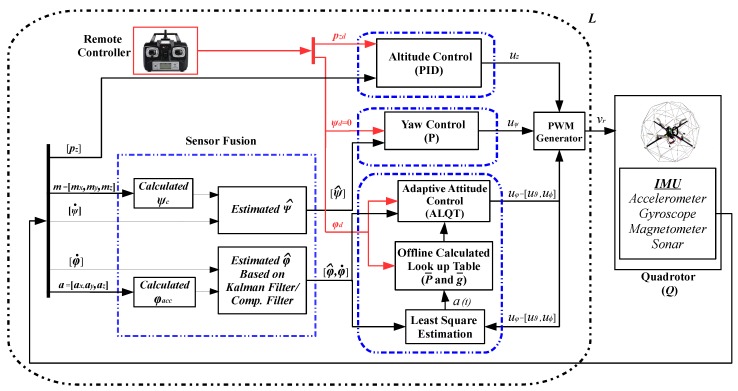
The overall quadrotor UAV control block diagram.

As studied in earlier work [4], we consider the quadrotor UAV control structure in two levels: high-level and low-level. High-level is mainly about guidance and position controlling in the autonomous motion tasks and generating the trajectories to be tracked by the low-level controller. Provided the trajectory from high-level, the low-level control is responsible for the quadrotor UAV’s attitude and altitude tracking performance and stability. In this study, we focus on the low-level control design, following a decentralized approach, considering the three motion dynamics modes separately: adaptive LQT control for the attitude dynamics, proportional (P) control for the yaw dynamics, and proportional-integral-derivative (PID) control for the altitude dynamics, as shown in Figure 1. In the overall structure, the attitude measurement noises, which come from IMU sensors, are compensated using a Kalman filter to obtain more reliable attitude estimation. The effectiveness of the employed Kalman filter is investigated over the experiments that compare the Kalman filter results with a complementary filter. In the next step, we developed an infinite-horizon ALQT controller and validated its effectiveness by performing two sets of experiments.

The rest of the paper is organized as follows: the quadrotor UAV system and the UAV attitude tracking problem are presented in Section 2. The filters are designed to fuse IMU data for reliable attitude parameter estimation are presented in Section 3. In Section 4 and Section 5, the ALQT, P and PID control designs (for attitude, yaw and altitude control, respectively) are developed. In Section 6, the proposed control schemes are tested on the experimental testbed and the test results are discussed. In Section 7, concluding remarks are given.

## 2. System Dynamics and Problem Definition

### 2.1. Quadrotor UAV Dynamics

A nonlinear dynamic model of quadrotor UAV motion dynamics is presented in [4]. In this paper, we have simplified and partitioned this nonlinear dynamic model to obtain separate linear models for each of attitude, yaw, and altitude dynamics.

#### 2.1.1. Attitude Model

Ignoring inertial and drag effects, we obtain a linearized attitude (roll/pitch) dynamics from the nonlinear dynamic model in [4]. Hence, we write the attitude model in the state-space form as

(1) $$\begin{array}{cc} {\dot{x}}_{\varphi }\left(t\right) & =A_{\varphi }x_{\varphi }\left(t\right)+B_{\varphi }u_{\varphi }\left(t\right),\;\;\varphi \in \left\{\vartheta ,\phi \right\},\\ \varphi (t) & =C_{\varphi }x_{\varphi }\left(t\right), \end{array}$$

where $A_{\varphi }=\left[\begin{array}{ccc} 0 & 1 & 0\\ 0 & 0 & \frac{2lK}{J_{\varphi }}\\ 0 & 0 & -b \end{array}\right]$, $B_{\varphi }=\left[\begin{array}{c} 0\\ 0\\ b \end{array}\right]$, $C_{\varphi }={\left[\begin{array}{c} 1\\ 0\\ 0 \end{array}\right]}^{T}$ and $x_{\varphi }=\left[\begin{array}{c} \varphi \\ \dot{\varphi }\\ T_{\varphi } \end{array}\right]$. $x_{\varphi }$, $u_{\varphi }$, φ, $\dot{\varphi }$, $T_{\varphi }$, $J_{\varphi }$, *K*, *b* and *l* represent states, control inputs, Euler angles, angular velocities, thrust forces, rotational inertias, positive armature gain, the actuator bandwidth in attitude (roll/pitch) dynamics and the distance between the center of gravity $O_{b}$ and each propeller, respectively.

**Remark** **1.** *Attitude (φ) dynamics represent roll (ϑ) and pitch (ϕ) dynamics, and yaw (ψ) is separated from attitude dynamics for the proposed control design.*

#### 2.1.2. Yaw Model

We obtain linearized yaw dynamic as

(2) $$\begin{array}{c} \ddot{\psi }=\frac{4K_{\psi }K}{J_{\psi }}\frac{b}{\left(s+b\right)}u_{\psi }, \end{array}$$

where $u_{\psi }$ is the yaw control input, $K_{\psi }$ is thrust-to-moment gain and $J_{\psi }$ is the rotational inertia in yaw motion. Finally, we write the linearized yaw model in form of an input–output transfer function as:

(3) $$\begin{array}{c} \psi =\frac{4K_{\psi }Kb}{s^{2}\left(s+b\right)J_{\psi }}u_{\psi }. \end{array}$$

#### 2.1.3. Altitude Model

We have linearized the nonlinear altitude model [4] by the use of small angle approximation and take the effect of gravity as an offset in the linearized model. Accordingly, we obtain the simplified linear altitude model as

(4) $$\begin{array}{c} {\ddot{p}}_{z}=\frac{4K}{m}\frac{b}{\left(s+b\right)}u_{z}, \end{array}$$

where $p_{z}$ is z-position of $O_{b}$, $u_{z}$ is the altitude control input and *m* is the total mass of the quadrotor UAV system. Finally, we obtain the linearized altitude model in the form of an input–output transfer function as

(5) $$\begin{array}{c} p_{z}=\frac{4Kb}{s^{2}\left(s+b\right)m}u_{z}. \end{array}$$

### 2.2. Problem Statement

Considering a quadrotor UAV with attitude (roll/pitch) dynamics (1), yaw dynamics (3), and altitude dynamics (5), as illustrated in Figure 1, the objectives of the paper are threefold:

1. Given the IMU sensor measurements of the attitude angles, design a data fusion algorithm based on (i) Kalman filtering and, for comparative analysis purposes, (ii) complementary filtering, in order to cancel the IMU sensor noise effects and produce accurate attitude state estimates;
2. Design the control units to generate the command signals $u_{z}$, $u_{\psi }$, $u_{\vartheta }$, $u_{\phi }$ for feeding the pulse width modulation (PWM) generator that generates the motor control input signal $v_{r}$, per the diagram in Figure 1: (a) design an infinite-horizon ALQT controller to generate the optimal attitude control signal $u_{\varphi }\left(t\right)=u_{\varphi }^{*}\left(t\right)$ so that φ(t) tracks its desired trajectory $\varphi_{d}\left(t\right)$, minimizing the predefined quadratic performance optimal tracking and energy consumption *cost function*
  (6) $$\begin{array}{c} J=\frac{1}{2}\int_{0}^{\infty }\left(Qe_{\varphi }^{2}\left(t\right)+Ru_{\varphi }^{2}\left(t\right)\right)dt, \end{array}$$
  where *Q* and *R* are positive constant weighting terms and
  (7) $$\begin{array}{c} e_{\varphi }\left(t\right)=\varphi \left(t\right)-\varphi_{d}\left(t\right) \end{array}$$
  is the attitude tracking *error*; (b) design a P yaw controller to generate $u_{\psi }\left(t\right)$ and a PID altitude controller to generate $u_{z}\left(t\right)$;
3. Combining the designs in 1 and 2, above, real-time implement and experimentally validate the overall control scheme.

### 2.3. Control Approach

In our infinite-horizon ALQT control design, the optimal control law consists of two terms: the state-feedback and the feed-forward. The state-feedback term maintains stability of the attitude dynamics. This term is obtained solving an algebraic Riccati equation (ARE). The feed-forward term depends on the desired trajectory and is used for establishing trajectory tracking performances. The above optimal control law is combined with an LS based adaptive PI algorithm to make it robust, adaptive and avoid inertial uncertainties in the attitude dynamics. After this combination, because of the uncertainties, the ARE needs to be solved online as well. In implementation, by comparing the online estimates of the uncertain parameters with some critical parameters calculated and stored in a look-up table, the time-varying state-feedback (from the PI) and then the time-varying feed-forward (from slowly-varying desired attitude and the PI) terms are calculated online. In real-time implementation of the designed ALQT scheme, we utilize a practical real-time computation technique based on parameterized analytical solutions of the state-feedback and the feed-forward terms.

## 3. IMU Sensor Data Fusion

**Figure 2:**
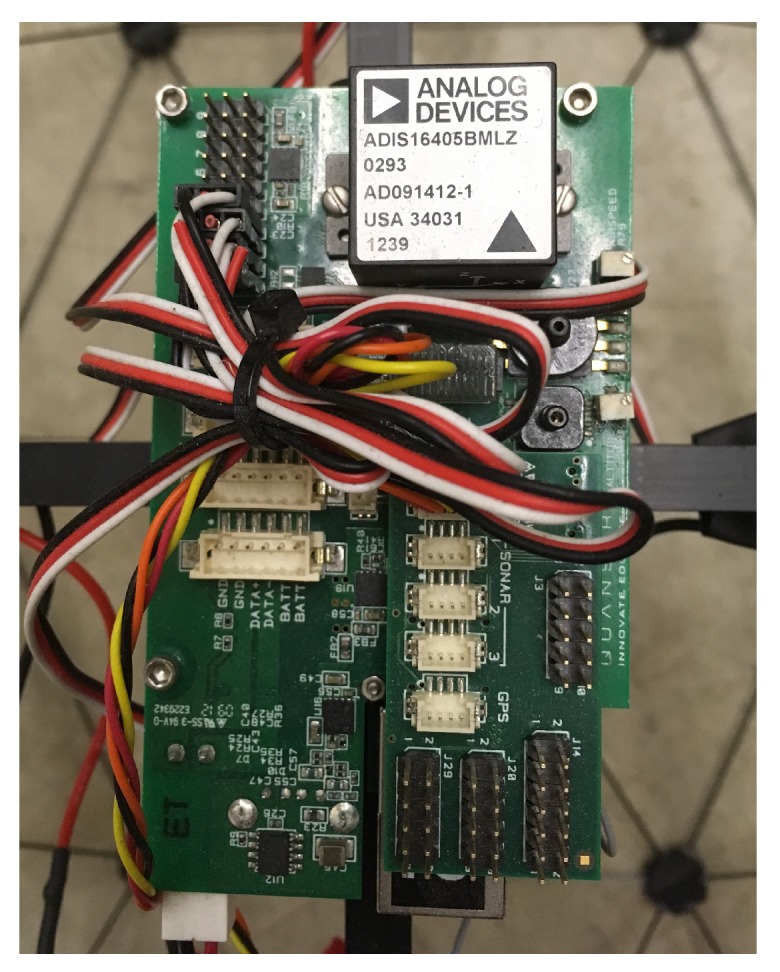
The ADIS16405 IMU module on the Qball-X4 quadrotor UAV.

The quadrotor UAV needs a robust estimation scheme for denoising the attitude angle measurements to provide reliable feedback to the proposed ALQT control scheme. The attitude angles are measured using an ADIS16405 IMU as shown in Figure 2. Then, a Kalman filter is employed to attenuate the effect of measurement noises. The IMU contains a 3-axis gyroscope to measure angular velocities $\left(\dot{\vartheta },\dot{\phi },\dot{\psi }\right)$, a 3-axis accelerometer to measure accelerations due to Earth’s gravity $\left(a_{x},a_{y},a_{z}\right)$ and a 3-axis magnetometer to measure the magnetic field intensities $\left(m_{x},m_{y},m_{z}\right)$. The specifications are listed in Table 1.

### 3.1. Attitude Determination from IMU Sensors

Roll and pitch angles are obtained based on accelerometer and gravity vector relation. The rotation matrix from the body frame to the inertial frame is defined with the Euler angles as:

(8) $$\begin{array}{cc} R_{b2i} & =\left[\begin{array}{ccc} \cos\psi \cos\vartheta & -\sin\psi \cos\phi +\cos\psi \sin\vartheta \sin\phi & \sin\psi \sin\phi +\cos\psi \sin\vartheta \cos\phi \\ \sin\psi \cos\vartheta & \cos\psi \cos\phi +\sin\psi \sin\vartheta \sin\phi & -\cos\psi \sin\phi +\sin\psi \sin\vartheta cos\phi \\ -\sin\vartheta & \cos\vartheta \sin\phi & \cos\vartheta \cos\phi \end{array}\right]. \end{array}$$

Assuming constant translational velocities [30,42], i.e., ignoring translational accelerations, we obtain the following relation between the accelerometer output, rotation matrix and earth gravity:

(9) $$\begin{array}{c} \left[\begin{array}{c} a_{x}\\ a_{y}\\ a_{z} \end{array}\right]=R_{i2b}\left[\begin{array}{c} 0\\ 0\\ g \end{array}\right]=\left[\begin{array}{c} -\sin\vartheta \\ \cos\vartheta \sin\phi \\ \cos\vartheta \cos\phi \end{array}\right]g, \end{array}$$

where $R_{i2b}=R_{b2i}^{T}$. From Equation (9), attitude angles are calculated as

(10) $$\begin{array}{c} \varphi_{acc}=\left[\begin{array}{c} \vartheta_{acc}\\ \phi_{acc} \end{array}\right]=\left[\begin{array}{c} \mathrm{atan}2(-a_{x},\sqrt{a_{y}^{2}+a_{z}^{2}})\\ \mathrm{atan}2(a_{y},a_{z}) \end{array}\right], \end{array}$$

where $\mathrm{atan}2(a_{y},a_{z})$ denotes arc tangent of $a_{x}$ and $a_{y}$ while it uses the signs of both arguments to determine the quadrant of the result. By determining the roll and pith angles, the rotation matrix from the body frame to the magnetometer local (NED:North-East-Down) frame is rearranged as

(11) $$\begin{array}{c} \left[\begin{array}{c} m_{x}\\ m_{y}\\ m_{z} \end{array}\right]=\left[\begin{array}{ccc} \cos\vartheta & \sin\vartheta \sin\phi & \sin\vartheta \cos\phi \\ 0 & \cos\phi & -\sin\phi \\ -\sin\vartheta & \cos\vartheta \sin\phi & \cos\vartheta \cos\phi \end{array}\right]\left[\begin{array}{c} m_{xb}\\ m_{yb}\\ m_{zb} \end{array}\right]. \end{array}$$

Hence, yaw (heading) is calculated as

(12) $$\begin{array}{c} \psi_{c}=\left[\begin{array}{c} \mathrm{atan}2(m_{y},m_{x}) \end{array}\right]. \end{array}$$

In practice, the yaw (heading) is updated by gyroscope data integration instead of a Kalman filter or a complementary filter since the laboratory environment has magnetic (metallic) disturbances on the heading calculation (12). Solution methods of magnetic disturbances on heading calculation are discussed with the details in [43].

### 3.2. Attitude Estimation Using a Kalman Filter

To filter IMU accelerometer noises, a linear Kalman filter is employed in this paper. At each time step *k*, this Kalman filter first predicts the state propagation using the dynamic model of the quadrotor UAV, the control inputs applied at step k−1 and the state measurement at step k−1. Then, it incorporates new measurement data of step *k*, to determine the state estimate.

Consider the following discrete-time linear time-invariant model of the attitude dynamics, with additive Gaussian measurement noise and disturbance, based on Equation (1):

(13) $$\begin{array}{cc} x[k+1] & =A_{d}x\left[k\right]+B_{d}u\left[k\right]+w, \end{array}$$

(14) $$\begin{array}{cc} y[k] & =C_{d}x\left[k\right]+v, \end{array}$$

where *w* is zero mean Gaussian disturbance noise with covariance $Q_{K}$, *v* is zero mean Gaussian measurement noise with covariance $R_{K}$, and

(15) $$\begin{array}{c} A_{d}=\left[\begin{array}{ccc} 1 & T_{s} & 0\\ 0 & 1 & \frac{2lK}{J_{\varphi }}T_{s}\\ 0 & 0 & 1-bT_{s} \end{array}\right],B_{d}=\left[\begin{array}{c} 0\\ 0\\ bT_{s} \end{array}\right],C_{d}=\left[\begin{array}{ccc} 1 & 0 & 0\\ 0 & 1 & 0 \end{array}\right], \end{array}$$

with sampling time $T_{s}$. Note that, in implementation of Equation (15), since the value of the rotational inertia $J_{\varphi }$ is uncertain, the nominal value of this parameter is used, as detailed in Remark 2 in Section 4.1. For this system model, the Kalman filter prediction and update equations are as follows:

Prediction:

(16) $$\begin{array}{cc} \hat{x}\left[k+1|k\right] & =A_{d}x\left[k|k\right]+B_{d}u\left[k\right], \end{array}$$

(17) $$\begin{array}{cc} P[k+1|k] & =A_{d}P\left[k|k\right]A_{d}^{T}+Q_{K}, \end{array}$$

Update:

(18) $$\begin{array}{cc} \bar{y}\left[k\right] & =y\left[k\right]-C_{d}\hat{x}\left[k|k-1\right], \end{array}$$

(19) $$\begin{array}{cc} M[k] & =P\left[k|k-1\right]C_{d}^{T}{\left(C_{d}P\left[k|k-1\right]C_{d}^{T}+R_{K}\right)}^{-1}, \end{array}$$

(20) $$\begin{array}{cc} \hat{x}\left[k|k\right] & =\hat{x}\left[k|k-1\right]+M\left[k\right]\bar{y}\left[k\right], \end{array}$$

(21) $$\begin{array}{cc} P[k|k] & =(I-M\left[k\right]C_{d})P\left[k|k-1\right], \end{array}$$

where P[k+1|k] and M[k] are the predicted error covariance and the optimal Kalman gain, respectively.

### 3.3. Attitude Estimation by a Complementary Filter

**Figure 3:**
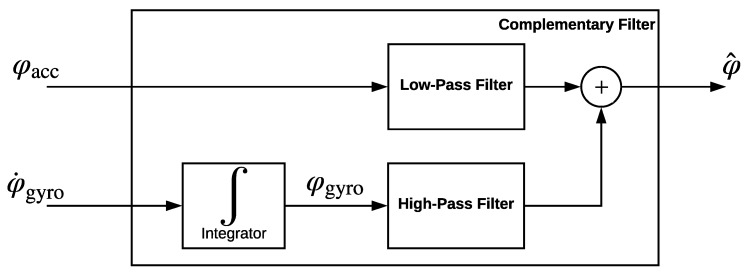
Complementary filter.

As an alternative to Kalman filtering, we also study utilization of complementary filter in denoising and fusion of measurement data from accelerometers and gyroscopes. Typically, an accelerometer based orientation estimation works better in static conditions, and, on the other hand, a gyroscope based orientation estimation gives better results in dynamic conditions. A complementary filter passes the accelerometer signals through a low-pass filter and the gyroscope signals integral through a high-pass filter. Then, the resulting signals are summed up to estimate the attitude angles more reliably in both dynamic and static condition cases. The schematic complementary filter block diagram is depicted in Figure 3.

## 4. Adaptive Optimal Attitude Tracking Control Design

In this section, the proposed ALQT control scheme for attitude tracking of a quadrotor UAV is presented.

### 4.1. Adaptive Parameter Identification Scheme

We employ an LS based PI scheme to estimate the uncertain inertial parameters. From the attitude dynamics Equation (1), following the procedure in [44,45], we first define a linear parametric model avoiding need for signal differentiation and the associated noise sensitivity issue by use of the stable filter $\frac{1}{\left(s+\lambda \right)},\;\lambda >0,$ as follows:

(22) $$\begin{array}{cc} z_{\varphi } & =\theta_{\varphi }^{*}\Phi_{\varphi },\\ \;z_{\varphi }=\frac{s}{\left(s+\lambda \right)}\dot{\varphi },\theta_{\varphi }^{*} & =\frac{1}{J_{\varphi }},\Phi_{\varphi }=\frac{2lKb}{\left(s+\lambda )(s+b\right)}u_{\varphi }, \end{array}$$

noting that the Euler rate $\dot{\varphi }$ (obtained using the IMU and the filters in Section 3) and the control signal $u_{\varphi }$ are measurable, and l,K,b are known constant parameters.

**Assumption** **1.** The upper and lower limits of $\theta_{\varphi }^{*}\left(t\right)$ are known, i.e., $0<{\underline{\theta }}_{\varphi }\leq \theta_{\varphi }^{*}\left(t\right)\leq {\bar{\theta }}_{\varphi }$ for some known ${\underline{\theta }}_{\varphi },{\bar{\theta }}_{\varphi }>0$.

**Remark** **2.** *For the setup used in this paper, the limits of ${\hat{\theta }}_{\varphi }\left(t\right)$ are taken $10\leq {\hat{\theta }}_{\varphi }\left(t\right)\leq 49$. Accordingly, the nominal value of $J_{\varphi }$ is calculated as $J_{\varphi 0}=\frac{2}{{\underline{\theta }}_{\varphi }+{\bar{\theta }}_{\varphi }}\approx 0.03$.*

To generate the estimate ${\hat{\theta }}_{\varphi }$ of the uncertain inertia parameter $\theta_{\varphi }^{*}$, we apply the following recursive LS algorithm [45] based on the parametric model (22):

(23) $$\begin{array}{cc} {\dot{\hat{\theta }}}_{\varphi }\left(t\right)=Pr\left(p\left(t\right)\epsilon \left(t\right)\Phi_{\varphi }\left(t\right)\right) & =\left\{\begin{array}{cc} p\left(t\right)\epsilon \left(t\right)\Phi_{\varphi }\left(t\right),\;\;\;\; & \mathrm{if}\;{\underline{\theta }}_{\varphi }<{\hat{\theta }}_{\varphi }<{\bar{\theta }}_{\varphi }\\ 0,\;\;\;\;\;\;\;\;\;\;\;\;\;\;\;\;\;\;\;\;\;\;\;\;\;\;\;\; & \mathrm{otherwise}, \end{array}\right.,\;\;\;\;\;{\hat{\theta }}_{\varphi }\left(0\right)={\hat{\theta }}_{\varphi 0},\\ \dot{p}\left(t\right) & =\left\{\begin{array}{cc} \beta p\left(t\right)-\frac{\Phi_{\varphi }^{2}\left(t\right)}{m_{n}^{2}\left(t\right)}p^{2}\left(t\right), & \mathrm{if}\;{\underline{\theta }}_{\varphi }<{\hat{\theta }}_{\varphi }<{\bar{\theta }}_{\varphi }\\ 0,\;\;\;\;\;\;\;\;\;\;\;\;\;\;\;\;\;\;\;\;\; & \mathrm{otherwise}, \end{array}\right.,\\ \epsilon (t) & =\frac{z_{\varphi }\left(t\right)-{\hat{\theta }}_{\varphi }\left(t\right)\Phi_{\varphi }\left(t\right)}{m_{n}^{2}\left(t\right)},\;\;\;m_{n}^{2}\left(t\right)=1+\alpha_{n}\Phi_{\varphi }^{2}\left(t\right),\;\;1\gg \alpha_{n}>0, \end{array}$$

where p(t) is the positive covariance (time varying gain) term with $p\left(0\right)=p_{0}>0$, $m_{n}$ is the normalizing signal, and ϵ is the estimation error. Pr(.) is projection operator that maintains ${\hat{\theta }}_{\varphi }\in \left[{\underline{\theta }}_{\varphi },{\bar{\theta }}_{\varphi }\right]$.

**Lemma** **1** (Stability and Convergence). Consider the LS based PI scheme (23), applied to the attitude dynamics (1). It is guaranteed that all the signals in the PI scheme (23), including p and $p^{-1}$, are bounded and ${\hat{\theta }}_{\varphi }\in \left[{\underline{\theta }}_{\varphi },{\bar{\theta }}_{\varphi }\right]$. Furthermore, if $\Phi_{\varphi n}=\frac{\Phi_{\varphi }}{m_{n}}$ is persistently exciting, i.e., if $\frac{1}{T}\int_{t}^{t+T}\Phi_{\varphi n}^{2}d\tau \geq \alpha_{0}$ for all t≥0 and some $T,\alpha_{0}>0$; then the PI scheme (23) ensures that $\theta_{\varphi }\left(t\right)\to \theta_{\varphi }^{*}$ as t→∞. The convergence of $\theta_{\varphi }\left(t\right)\to \theta_{\varphi }^{*}$ is exponential for β>0.

**Proof.**  The result is a direct corollary of the more general Theorem 3.7.4 and 3.10.1 in [45]. ☐

### 4.2. Generic Linear Quadratic Tracking Control Design

To construct the base optimal control law of the proposed ALQT scheme, we follow an infinite-horizon LQT control design approach [46], explained in the sequel for a linear system in the generic state-space form

(24) $$\begin{array}{cc} \dot{x}\left(t\right) & =Ax(t)+Bu(t),\\ y(t) & =Cx(t), \end{array}$$

where $x\in {ℜ}^{n}$, $u\in {ℜ}^{r}$ and $y\in {ℜ}^{m}$ are state, control input and output vectors. $A\in {ℜ}^{n\times n}$, $B\in {ℜ}^{n\times r}$ and $C\in {ℜ}^{m\times n}$ are state, input and output matrices. *m*, *n*, and *r* are generic system dimensions. The objective is to generate u(t) so that y(t) tracks a given desired continuous and differentiable output trajectory $z\left(t\right)\in {ℜ}^{m}$ as close as possible with minimum consumption of control effort for all *t*. Thus, let us define the *error vector*

(25) $$\begin{array}{c} e(t)=z(t)-y(t), \end{array}$$

and the *cost function*

(26) $$\begin{array}{c} J=\frac{1}{2}\int_{0}^{\infty }\left(e^{T}\left(t\right)Qe\left(t\right)+u^{T}\left(t\right)Ru\left(t\right)\right)dt, \end{array}$$

where $Q\in {ℜ}^{m\times m}$ and $R\in {ℜ}^{r\times r}$ are symmetric, positive definite weighting matrices.

In order to generate the optimal control signal $u\left(t\right)=u^{*}\left(t\right)$ that minimizes the cost function (26), following *Hamiltonian* calculation [46], at first, the following DRE is formed:

(27) $$\begin{array}{c} \dot{P}=-PA-A^{T}P+PBR^{-1}B^{T}P-C^{T}QC, \end{array}$$

where $P\in {ℜ}^{n\times n}$ is a symmetric, positive definite matrix. Since the infinite-horizon LQT design [46] is studied, there is no terminal $F(t_{f})=0$ in *cost function* (26). Therefore, P(t) tends to its steady-state value $\lim_{t_{f}\to \infty }\left(P\left(t_{f}\right)\right)=\bar{P}$ as the solution of the following ARE:

(28) $$\begin{array}{c} -\bar{P}A-A^{T}\bar{P}+\bar{P}BR^{-1}B^{T}\bar{P}-C^{T}QC=0, \end{array}$$

where $\bar{P}\in {ℜ}^{n\times n}$ is a symmetric, positive definite matrix calculated by analytical solution of the ARE (28).

Then, as the next step in the LQT design steps with a *Hamiltonian* approach, a vector signal $g\left(t\right)\in {ℜ}^{n}$ is generated via the differential equation

(29) $$\begin{array}{c} \dot{g}\left(t\right)=-\left[A^{T}-PBR^{-1}B^{T}\right]g\left(t\right)-C^{T}Qz\left(t\right). \end{array}$$

The final optimal control signal is generated as

(30) $$\begin{array}{c} u^{*}\left(t\right)=-R^{-1}B^{T}\bar{P}x\left(t\right)+R^{-1}B^{T}g\left(t\right), \end{array}$$

where $-R^{-1}B^{T}\bar{P}x\left(t\right)$ is the state feedback term and $R^{-1}B^{T}g\left(t\right)$ is the feed-forward term. Note that the control law (30) is established in [46] to bear the following optimal tracking property:

**Proposition** **1.** *Ref. [46]: The control law (30) guarantees that the system (24) is closed loop stable and the cost function (26) is minimized, for any given slowly-varying desired output trajectory z(t).*

To simplify and ease the calculation of the vector signal g(t), we use an approximation [47] as follows:

***Approximate vector signal*$\bar{g}\left(t\right)$***:* It is established in [47] that, if z(·) is slowly varying, then $\dot{g}\left(t\right)$ in Equation (29) can be approximated as $\dot{g}\left(t\right)\approx 0$ leading to the approximate solution

(31) $$\begin{array}{c} g\left(t\right)\approx \bar{g}\left(t\right)={\left[A^{T}-\bar{P}BR^{-1}B^{T}\right]}^{-1}\left[-C^{T}Qz\left(t\right)\right]. \end{array}$$

### 4.3. Adaptive Linear Quadratic Tracking (ALQT) Control Design

For attitude control, our approach is to apply the control law (28), (30), (31) to system (1). Note that implementation of the control law (30) requires $\bar{P}$ from Equation (28) and $\bar{g}\left(t\right)$ from Equation (31), and hence requires knowledge of the system matrices A,B,C. In our case, in Equation (1), although $B_{\varphi },C_{\varphi }$ are known, $A_{\varphi }$ is unknown. Hence, following the certainty equivalence approach [45,46], the following adaptive version of the LQT control law (28), (30), (31) for the *cost function* (6) and the attitude tracking *error* (7) is designed.

The time-varying adaptive ARE, the approximate vector signal $\bar{g}\left(t\right)$ and the adaptive optimal control signal are obtained, respectively, as

(32) $$\begin{array}{cc} -\bar{P}{\hat{A}}_{\varphi }\left(t\right) & -{\hat{A}}_{\varphi }^{T}\left(t\right)\bar{P}+\bar{P}B_{\varphi }R^{-1}B_{\varphi }^{T}\bar{P}-C_{\varphi }^{T}QC_{\varphi }=0, \end{array}$$

(33) $$\begin{array}{cc} \bar{g}\left(t\right) & ={\left[{\hat{A}}_{\varphi }^{T}\left(t\right)-\bar{P}B_{\varphi }R^{-1}B_{\varphi }^{T}\right]}^{-1}\left[-C_{\varphi }^{T}Qz\left(t\right)\right], \end{array}$$

(34) $$\begin{array}{cc} {\hat{u}}_{\varphi }^{*}\left(t\right) & =-R^{-1}B_{\varphi }^{T}\bar{P}x_{\varphi }\left(t\right)+R^{-1}B_{\varphi }^{T}\bar{g}\left(t\right), \end{array}$$

where ${\hat{A}}_{\varphi }\left(t\right)=\left[\begin{array}{ccc} 0 & 1 & 0\\ 0 & 0 & 2lK{\hat{\theta }}_{\varphi }\left(t\right)\\ 0 & 0 & -b \end{array}\right]$, $B_{\varphi }=\left[\begin{array}{c} 0\\ 0\\ b \end{array}\right]$, $C_{\varphi }={\left[\begin{array}{c} 1\\ 0\\ 0 \end{array}\right]}^{T}$. Solving (32) for $\bar{P}=\left[\begin{array}{ccc} {\bar{P}}_{1} & {\bar{P}}_{2} & {\bar{P}}_{3}\\ {\bar{P}}_{2} & {\bar{P}}_{4} & {\bar{P}}_{5}\\ {\bar{P}}_{3} & {\bar{P}}_{5} & {\bar{P}}_{6} \end{array}\right]\in {ℜ}^{3\times 3},$ we obtain

(35a) $$\begin{array}{cc} 0 & =-({\bar{P}}_{3}^{2}b^{2}/R)+Q, \end{array}$$

(35b) $$\begin{array}{cc} 0 & =-{\bar{P}}_{1}+\left(\left({\bar{P}}_{3}{\bar{P}}_{5}b^{2}\right)/R\right), \end{array}$$

(35c) $$\begin{array}{cc} 0 & =-\left(2lK{\hat{\theta }}_{\varphi }{\bar{P}}_{2}\right)+{\bar{P}}_{3}b+\left({\bar{P}}_{3}{\bar{P}}_{6}b^{2}/R\right), \end{array}$$

(35d) $$\begin{array}{cc} 0 & =-2{\bar{P}}_{2}+\left({\bar{P}}_{5}^{2}b^{2}/R\right), \end{array}$$

(35e) $$\begin{array}{cc} 0 & =-\left(2lK{\hat{\theta }}_{\varphi }{\bar{P}}_{4}\right)+{\bar{P}}_{5}b-{\bar{P}}_{3}+\left({\bar{P}}_{5}{\bar{P}}_{6}b^{2}/R\right), \end{array}$$

(35f) $$\begin{array}{cc} 0 & =-\left(4lK{\hat{\theta }}_{\varphi }{\bar{P}}_{5}\right)-2{\bar{P}}_{6}b+\left({\bar{P}}_{6}^{2}b^{2}/R\right). \end{array}$$

Solving Equation (33) for $\bar{g}\left(t\right)={\left[{\bar{g}}_{1}\left(t\right)\;{\bar{g}}_{2}\left(t\right)\;{\bar{g}}_{3}\left(t\right)\right]}^{T}\in {ℜ}^{3}$, we obtain

(36a) $$\begin{array}{cc} {\bar{g}}_{1}\left(t\right) & =\left[\left({\bar{P}}_{5}Q\right)/\left({\bar{P}}_{3}R\right)\right]\varphi_{d}\left(t\right), \end{array}$$

(36b) $$\begin{array}{cc} {\bar{g}}_{2}\left(t\right) & =\left[\left({\bar{P}}_{6}bQ+RQ\right)/\left(2lK{\hat{\theta }}_{\varphi }{\bar{P}}_{3}\right)\right]\varphi_{d}\left(t\right), \end{array}$$

(36c) $$\begin{array}{cc} {\bar{g}}_{3}\left(t\right) & =\left[\left(RQ\right)/\left({\bar{P}}_{3}b^{2}\right)\right]\varphi_{d}\left(t\right). \end{array}$$

**Remark** **3.** *There is also no constraint on the control signal ${\hat{u}}_{\varphi }\left(t\right)$ considered in the control design. However, we consider a limit $-0.025⩽u_{\varphi }\left(t\right)⩽0.025$ to prevent damages on the quadrotor motors due to high torque commands.*

## 5. Yaw and Altitude Control

To provide the overall motion in experiments, P and PID controllers are designed, respectively, for yaw and altitude dynamics as follows.

### 5.1. Yaw Control

Since yaw dynamics are not directly affecting the lateral motion of the quadrotor UAV system, the yaw motion control is considered independently. Therefore, the following P control law is used based on the dynamic model (3):

(37) $$\begin{array}{cc} u_{\psi } & =K_{p\psi }e_{\psi }, \end{array}$$

where $e_{\psi }=\left(\psi_{d}-\psi \right)$.

### 5.2. Altitude Control

Altitude controller is derived for keeping the quadrotor UAV system in its desired altitude and providing stability at the longitudinal motion. The following PID control law is used based on the dynamic model (5):

(38) $$\begin{array}{c} u_{z}=K_{pz}\left(e_{z}\right)+K_{iz}\int_{0}^{t}\left(e_{z}\right)dt+K_{dz}\left({\dot{e}}_{z}\right), \end{array}$$

where $e_{z}=\left(p_{zd}-p_{z}\right)$.

**Remark** **4.** With the attitude, yaw and altitude control schemes as designed above, the control inputs ${\hat{u}}_{\varphi }^{*},\;u_{\psi }$ and $u_{z}$ are generated. Then, we combine these inputs [4] to generate each motor PWM inputs $v_{r}$. for flight control of the Qball-X4 quadrotor UAV system (Quanser, Markham, ON, Canada).

## 6. Experimental Tests and Comparative Simulations

### 6.1. Test Platform

**Figure 4:**
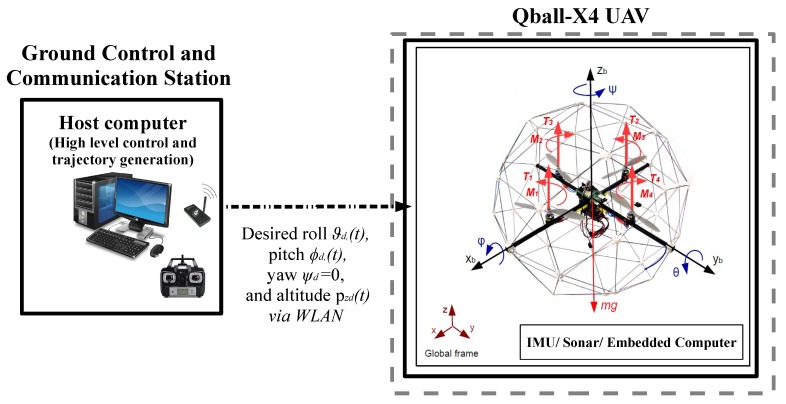
The Qball-X4 quadrotor UAV test platform.

The test platform consists of a Qball-X4 quadrotor UAV and a ground control and communication station (host computer) as illustrated in Figure 4. The Qball-X4 is equipped with a sonar sensor and an IMU to provide altitude, acceleration, angular rate and magnetometer measurements [48]. It has an on-board avionics data acquisition card (DAQ) and a Gumstix embedded computer (Gumstix Inc., Redwood City, CA, USA) for interfacing with on-board sensors and driving the four rotor motors. Each motor is linked to one of the four PWM servo output channels on the DAQ. The Qball-X4 dynamic parameters as specified in [48] are presented in Table 2. The ground station computer is used for coding the designed control algorithms, and embedding on the Qball-X4 on-board computers before tests as well as generating the high-level control inputs in the form of desired attitude and altitude trajectories online during the tests. For control algorithm coding and embedding, Quarc, a MATLAB/Simulink^®^ based interface software developed by Quanser Inc., is used.

### 6.2. Control Design Specifications and Online Calculation of Control Parameters

In implementation of the ALQT control design explained in detail in Section 4.3, the error and the control weighting parameters are chosen as Q=100 and R=30,000. Following Equation (35a), the constant entry ${\bar{P}}_{3}$ of $\bar{P}\left(t\right)$ is calculated as ${\bar{P}}_{3}=\sqrt{\left(QR\right)}/b=115.4701$. The other entries of $\bar{P}\left(t\right)$ are calculated solving Equation (35) online, noting the dependence of these entries to each other and the parameter estimate ${\hat{\theta }}_{\varphi }$. From Equation (35d), the entry ${\bar{P}}_{2}$ is found in form of the entries ${\bar{P}}_{5}$ and written in Equation (35c). Then, the Equations (35c) and (35f) are obtained in form of the entries ${\bar{P}}_{5}$ and ${\bar{P}}_{6}$ as follows:

(39a) $$\begin{array}{cc} 0 & =-\left(lK{\hat{\theta }}_{\varphi }{\bar{P}}_{5}^{2}b^{2}/R\right)+{\bar{P}}_{3}b+\left({\bar{P}}_{3}{\bar{P}}_{6}b^{2}/R\right), \end{array}$$

(39b) $$\begin{array}{cc} 0 & =-\left(4lK{\hat{\theta }}_{\varphi }{\bar{P}}_{5}\right)-2{\bar{P}}_{6}b+\left({\bar{P}}_{6}^{2}b^{2}/R\right). \end{array}$$

**Figure 5:**
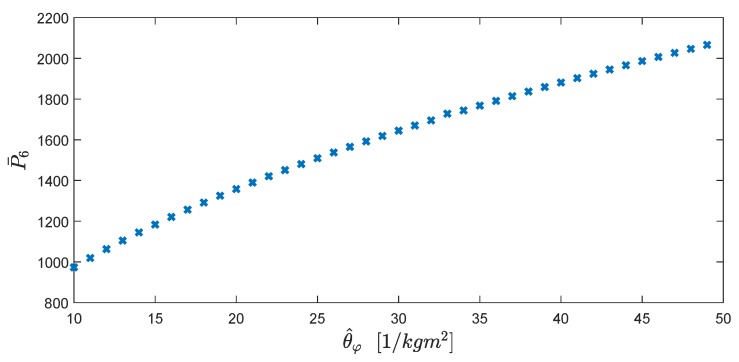
Offline calculation of ${\bar{P}}_{6}$ for the estimate ${\hat{\theta }}_{\varphi }\in \left[{\underline{\theta }}_{\varphi },{\bar{\theta }}_{\varphi }\right]$.

Solving the Equations (39a) and (39b) by using Maple^®^ and MATLAB^®^ software, ${\bar{P}}_{6}$, which is chosen as a critical parameter, is offline calculated for the estimate ${\hat{\theta }}_{\varphi }\in \left[{\underline{\theta }}_{\varphi },{\bar{\theta }}_{\varphi }\right]$ of $\theta_{\varphi }^{*}$. Then, a lookup table is prepared as plotted in Figure 5. The remaining entries of $\bar{P}$ are simultaneously calculated using ${\bar{P}}_{6}$ and the estimate ${\hat{\theta }}_{\varphi }$ as follows:

(40a) $$\begin{array}{cc} {\bar{P}}_{5} & =\left(\left(2{\bar{P}}_{6}b\right)+\left(\left({\bar{P}}_{6}^{2}b^{2}\right)/R\right)\right)/\left(4lK{\hat{\theta }}_{\varphi }\right), \end{array}$$

(40b) $$\begin{array}{cc} {\bar{P}}_{2} & =\left({\bar{P}}_{5}^{2}b^{2}\right)/\left(2R\right), \end{array}$$

(40c) $$\begin{array}{cc} {\bar{P}}_{1} & =({\bar{P}}_{3}{\bar{P}}_{5}b^{2})/R, \end{array}$$

(40d) $$\begin{array}{cc} {\bar{P}}_{4} & =\left(\left(-{\bar{P}}_{3}\right)/2lK{\hat{\theta }}_{\varphi }\right)+\left(\left({\bar{P}}_{5}b\right)/2lK{\hat{\theta }}_{\varphi }\right)+\left(\left({\bar{P}}_{5}{\bar{P}}_{6}b^{2}\right)/2lK{\hat{\theta }}_{\varphi }R\right). \end{array}$$

After obtaining $\bar{P}$, by Equation (36) and the reference input $\varphi_{d}\left(t\right)$, the vector signal $\bar{g}\left(t\right)$ is found at each time instant as well. For example, for the nominal value ${\hat{\theta }}_{\varphi 0}=33\;\left[1/kgm^{2}\right]$, the Riccati coefficient matrix $\bar{P}$ is obtained using Equations (39a), (39b), (40a), (40b), (40c) and (40d) as follows:

(41) $$\begin{array}{cc} \bar{P} & =\left[\begin{array}{ccc} 20.0886934 & 2.0177780 & 115.4700538\\ 2.01777802 & 480969.75 & 23.19642512\\ 115.470053 & 23.196425 & 1727.886987 \end{array}\right]. \end{array}$$

After that, the vector signal $\bar{g}\left(t\right)$ is found by Equations (41) and (36) as follows:

(42) $$\begin{array}{c} \bar{g}\left(t\right)=\left[\begin{array}{c} 20.08869343750145\;\varphi_{d}\left(t\right)\\ 2.017798199381859\;\varphi_{d}\left(t\right)\\ 115.4700538758503\;\varphi_{d}\left(t\right) \end{array}\right]. \end{array}$$

The ALQT control design with specifications above is used for pitch and roll control. For yaw tracking, a P controller is used with gain $K_{p\psi }=0.015$, and, for altitude tracking, a PID controller is used with gains $K_{pz}=0.006$, $K_{iz}=0.008$ and $K_{dz}=0.002$.

In implementation of the adaptive PI scheme (23), the forgetting factor, the initial covariance, and the initial parameter estimate, are selected as, respectively, β=0.001, $p_{0}=10^{5}$ and $\theta_{0}=10$ [1/kg m${}^{2}$]. In the Kalman filter implementation, $Q_{K}$ and $R_{K}$ matrices are taken as $Q_{K}=10^{-3}I_{3}$ and $R_{K}=2\times 10^{-4}I_{2}$. $A_{d}$, $B_{d}$ and $C_{d}$ matrices are numerically obtained for the nominal value of $J_{\varphi 0}=0.03$ [kg m${}^{2}$] and the sampling time $T_{s}=0.005$ as

$$\begin{array}{c} A_{d}=\left[\begin{array}{ccc} 1 & 0.005 & 0\\ 0 & 1 & 8\\ 0 & 0 & 0.925 \end{array}\right],B_{d}=\left[\begin{array}{c} 0\\ 0\\ 0.075 \end{array}\right],C_{d}=\left[\begin{array}{ccc} 1 & 0 & 0\\ 0 & 1 & 0 \end{array}\right]. \end{array}$$

For the complementary filter, $\varphi_{acc}$ is passed through a low pass filter with transfer function $G_{a}\left(s\right)=\left(20s+1\right)/\left(100s^{2}+20s+1\right)$, and $\dot{\varphi }$ is passed through a high pass filter with transfer function $G_{g}\left(s\right)=\left(100s\right)/\left(100s^{2}+20s+1\right)$.

### 6.3. Experimental Results

**Figure 6:**
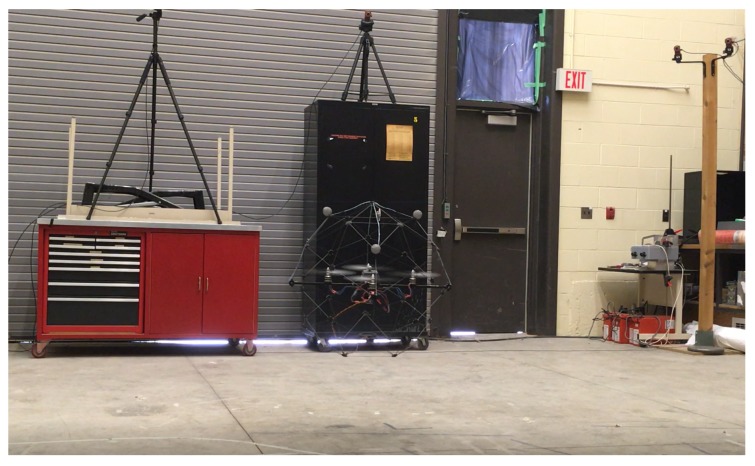
The Qball-X4 quadrotor during the experiment.

After setting all control parameters with the sampling rate 200 [Hz] by using MATLAB/Simulink^®^ and Quarc interface in the host computer, as explained in Section 6.1 and Section 6.2, we have implemented the proposed control scheme on the Qball-X4 as seen in Figure 6 for the following two cases:

Test 1. ALQT with complementary filter: The video of the experiment is presented in URL [49].
Test 2. ALQT with Kalman filter: The video of the experiment is presented in URL [50].

**Figure 7:**
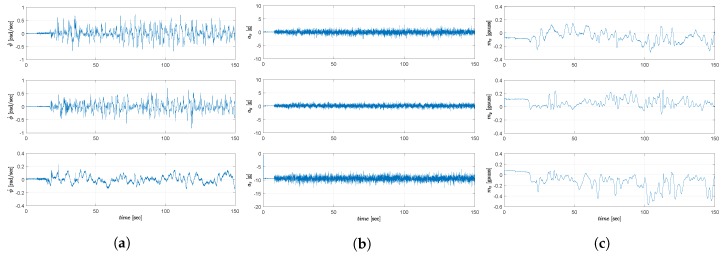
IMU data measurements from (**a**) gyroscope; (**b**) accelerometer; (**c**) magnetometer.

In both tests, the Qball-X4 starts to perform the tracking control task after hovering for 15 s. The real-time IMU data measurements in Test-2 from the gyroscope, the accelerometer and the magnetometer are presented in Figure 7a–c, respectively.

**Figure 8:**
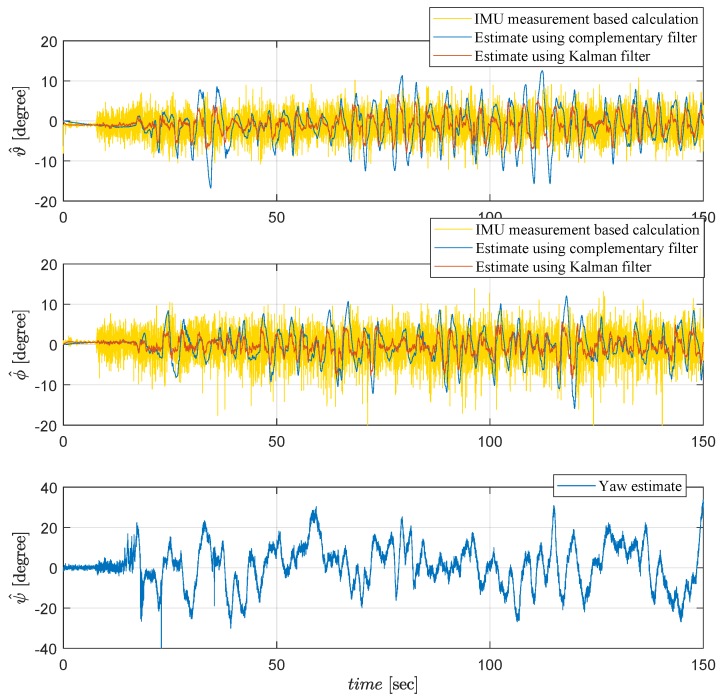
Attitude estimation of the Qball-X4.

**Figure 9:**
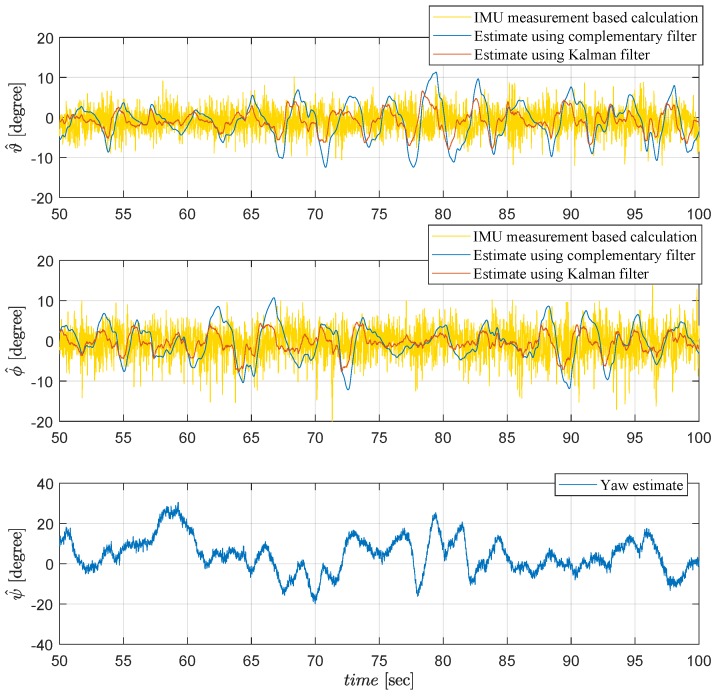
Attitude angle estimation of the Qball-X4 from 50 to 100 [s].

Applying the methodology explained in Section 3.1, the IMU measurements shown in Figure 6 are used to obtain the raw calculation of the roll and pitch parameters (yellow plots), and then to generate the estimates by complementary filter (blue plots) and Kalman filter (red plots) shown in Figure 8 and Figure 9. Kalman filter provides more reliable data less sensitive to noise. For the yaw estimation, gyroscope data integration is used instead, due to distortion effects by metallic objects of the test environment, as explained in Section 3.1.

**Figure 10:**
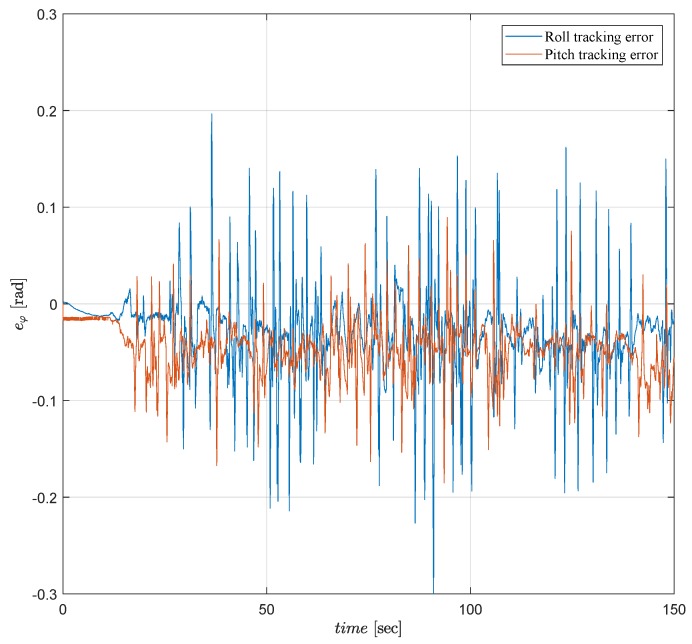
Attitude tracking error of the Qball-X4 using a Complementary filter.

**Figure 11:**
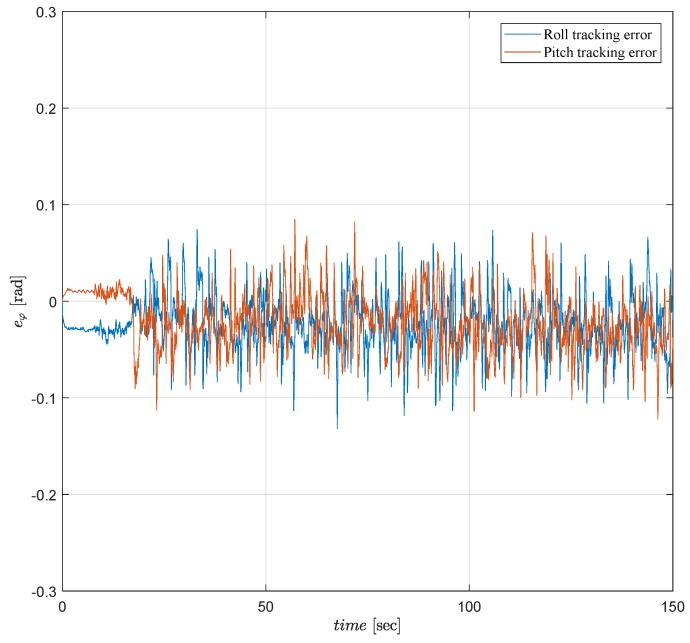
Attitude tracking error of the Qball-X4 using a Kalman filter.

The tracking error performances of both tests verify that the control objective is satisfied as seen in Figure 10 and Figure 11. In both tests, the controllers maintain attitude angles close to their desired angles with small attitude tracking errors ±0.1 [rad]. However, as seen in Figure 11, ALQT control with Kalman filter is more robust to sensor noises and uncertainties, and results in smaller tracking errors.

As seen in Table 3 and Table 4, ALQT control with Kalman filter gives significantly smaller mean-square error and consumes less battery (energy). It is also observed in additional simulations that the proposed controller consumes less battery energy with more robust control action compared to other classical controllers such as PID.

**Figure 12:**
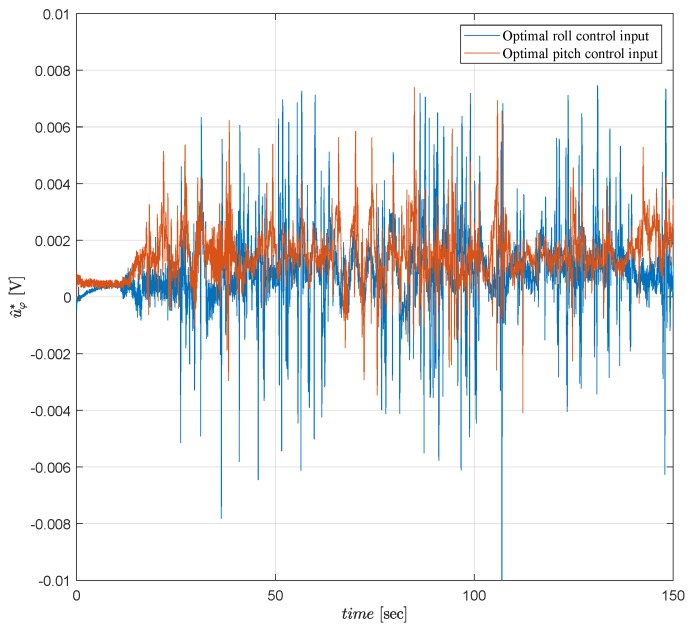
Optimal attitude control inputs for the complementary filter.

**Figure 13:**
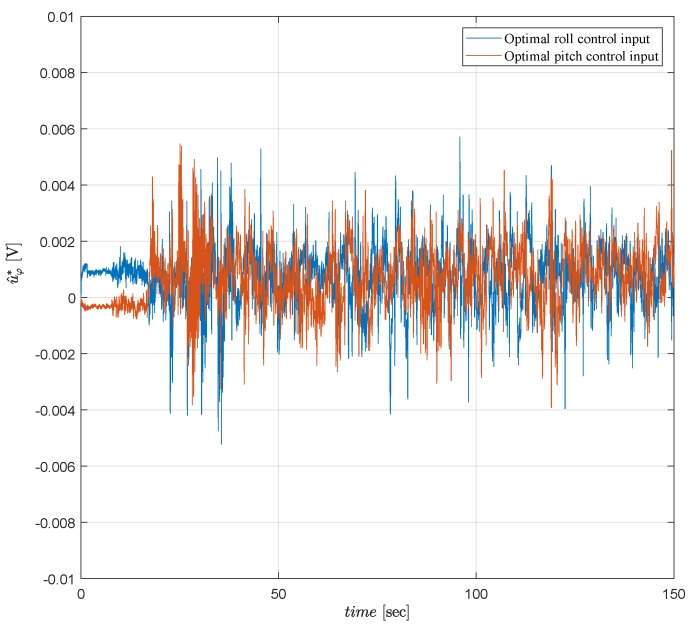
Optimal attitude control inputs for the Kalman filter.

**Figure 14:**
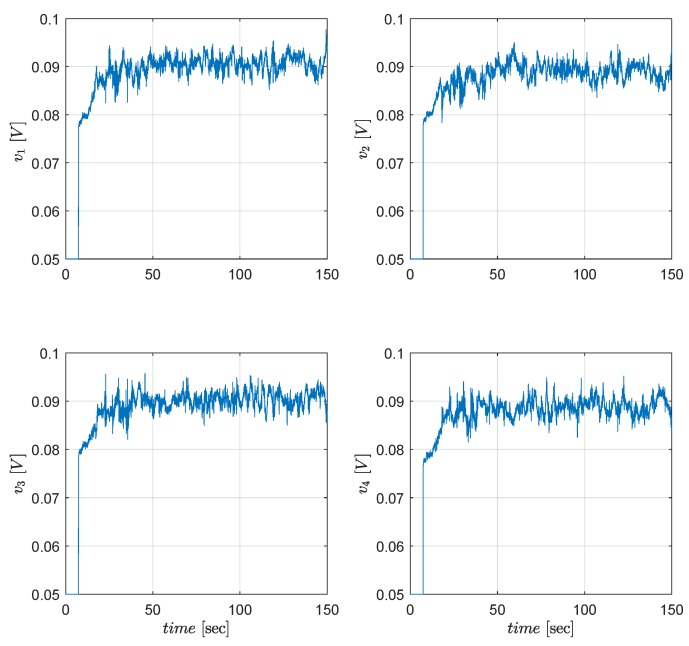
Motor PWM control inputs $v_{r}$.

In real time, the motor PWM control inputs have the constraint $-0.1⩽v_{r}\left(t\right)⩽0.1$ since they work with limited voltage to prevent damages due to high torque commands. Hence, a limit is applied for the optimal attitude control inputs as mentioned in Remark 3 even though LQT design procedure does not have any constraints. As seen in Figure 12, Figure 13 and Figure 14, the proposed controller satisfies admissible and optimal control actions for all t>0 during the tests. Figure 12, Figure 13 and Figure 14 show that the motor PWM and the optimal attitude control inputs are kept within the allowed limits.

**Figure 15:**
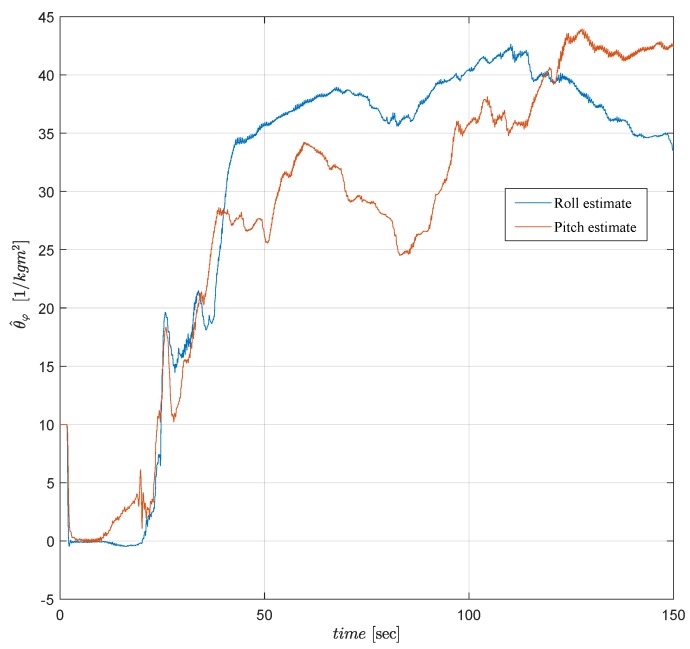
LS based estimate ${\hat{\theta }}_{\varphi }$ of the uncertain inertia parameter $\theta_{\varphi }^{*}$.

The LS based estimation of the uncertain inertia parameters ${\hat{\theta }}_{\vartheta }$ and ${\hat{\theta }}_{\phi }$ is presented in Figure 15. The estimates, which are purposely initialized at values away from the nominal values (to test the expected convergence), successfully converge to the vicinity of the nominal value 33 [1/kg m${}^{2}$] in around 40 [s]. Convergence rate of the estimation can be adjusted easily adjusting the design parameters of the LS based adaptive law.

### 6.4. Comparative Simulations and Observations

**Figure 16:**
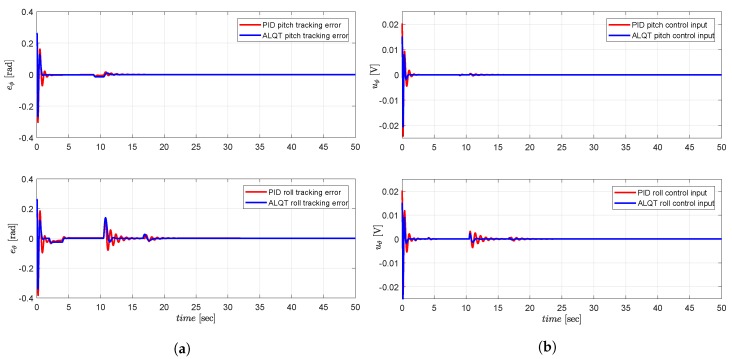
PID vs. ALQT performance comparison: (**a**) attitude tracking error; (**b**) attitude control input.

**Figure 17:**
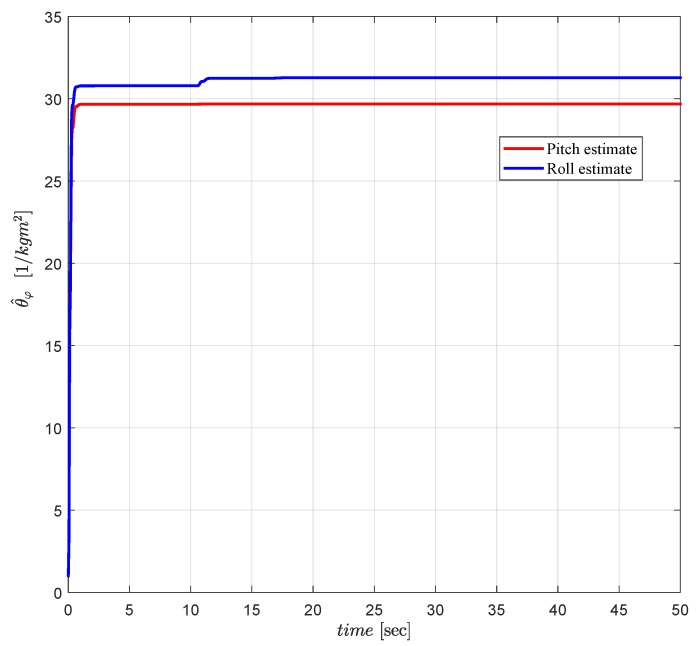
LS based estimate ${\hat{\theta }}_{\varphi }$ of the uncertain inertia parameter $\theta_{\varphi }^{*}$ for the simulation.

For optimal performance comparison with the existing literature, in ideal simulation conditions without noises, we simulate the ALQT control design and compare with a classical PID controller with control gains $K_{p}=0.0017$; $K_{i}=8.98$; $K_{d}=0.005$. As seen in Figure 16, the ALQT controller gives smaller tracking errors with less control action. Therefore, in the actual settings with noises, it is expected that the ALQT with a reliable filter will give us better tracking and control input performances compared to a PID controller. Figure 17 presents the estimates of the simulation. It is also observed from literature that the proposed controller gives a good control performance in terms of optimal attitude tracking compared to the attitude tracking errors of [11,12].

## 7. Conclusions

In this paper, the adaptive linear quadratic tracking (ALQT) scheme has been developed to control and stabilize the attitude of the Qball-X4 quadrotor UAV system in an optimal sense. The proposed adaptive controller is designed by an indirect approach and combined with the LS based parameter identification (PI) to eliminate the influences of inertial uncertainties. Additionally, the Kalman filter has been designed for canceling noise effects on the attitude estimation data to provide more reliable feedback to the controller and it is compared with the Complementary filter. All analytical analyses and designs are verified by the two experimental tests. We witness that the ALQT design in experiments work satisfactorily in terms of the optimal tracking performance. In the Kalman filter vs. Complementary filter, although both filter designs are good at canceling noise effects on the estimated attitude data, the Kalman filter gives a better accuracy and reliable attitude estimation. Thus, the experimental results show that the quadrotor UAV has more robust behavior and better tracking error with the estimated attitude data by the Kalman filter compared to the Complementary filter.

A potential future study is developing heading (yaw) estimation methods under magnetic disturbances. Another future direction is to extend optimal linear quadratic tracking control design for altitude and yaw dynamics.

## Figures and Tables

**Table 1:** The ADIS16405 IMU Specifications [41].

	Gyroscope	Accelerometer	Magnetometer
Range	±305 (deg/s)	±18 (g)	± 3.5 (gauss)
Sensitivity	0.05 (deg/s/LSB)	3.33 (mg/LSB)	0.5 (mgauss/LSB)

**Table 2:** The Qball-X4 quadrotor UAV dynamic parameters [48].

*m* (kg)	*l* (m)	*K* (N)	$K_{\psi }$ (Nm)	*b* (rad/s)	$J_{\varphi 0}$ (kg m${}^{2}$)	$J_{\psi }$ (kg m${}^{2}$)
1.4	0.2	120	4	15	0.03	0.04

**Table 3:** Mean square error of $e_{\varphi }$.

ALQT	Roll [rad]	Pitch [rad]
with Kalman filter	0.0012	0.0012
with comp. filter	0.0027	0.0029

**Table 4:** Average battery consumption by ${\hat{u}}_{\varphi }^{*}$.

ALQT	Roll [voltage/s]	Pitch [voltage/s]
with Kalman filter	0.00071	0.00066
with comp. filter	0.00086	0.00140
